# Supplementary material for: The tumor therapy landscape of synthetic lethality
Source: Nat Commun. 2021 Feb 24;12:1275. doi: 10.1038/s41467-021-21544-2 (PMC7904840; doi:10.1038/s41467-021-21544-2)
Supplement: Supplementary file 1 — Supplementary Information [file 41467_2021_21544_MOESM1_ESM.pdf]

Supplementary information

Supplementary Fig. 1. Boxplot and Bipartite.

Supplementary Fig. 2. Knockdown efficiency of *CDKN2A* was determined by Western blot.

Supplementary Table 1. Literature supports for the top 20 repurposable drug ca ndidates.

Supplementary Table 2. Clinical trial records of the top repurposable drug candidates based on SL and SDL respectively.

Supplementary Table 3. Best repurposable drug candidates of SL and SDL.

Supplementary Table 4. Mapping between tumors and cancer types.

Supplementary Table 5. Primer sequences used in this study.

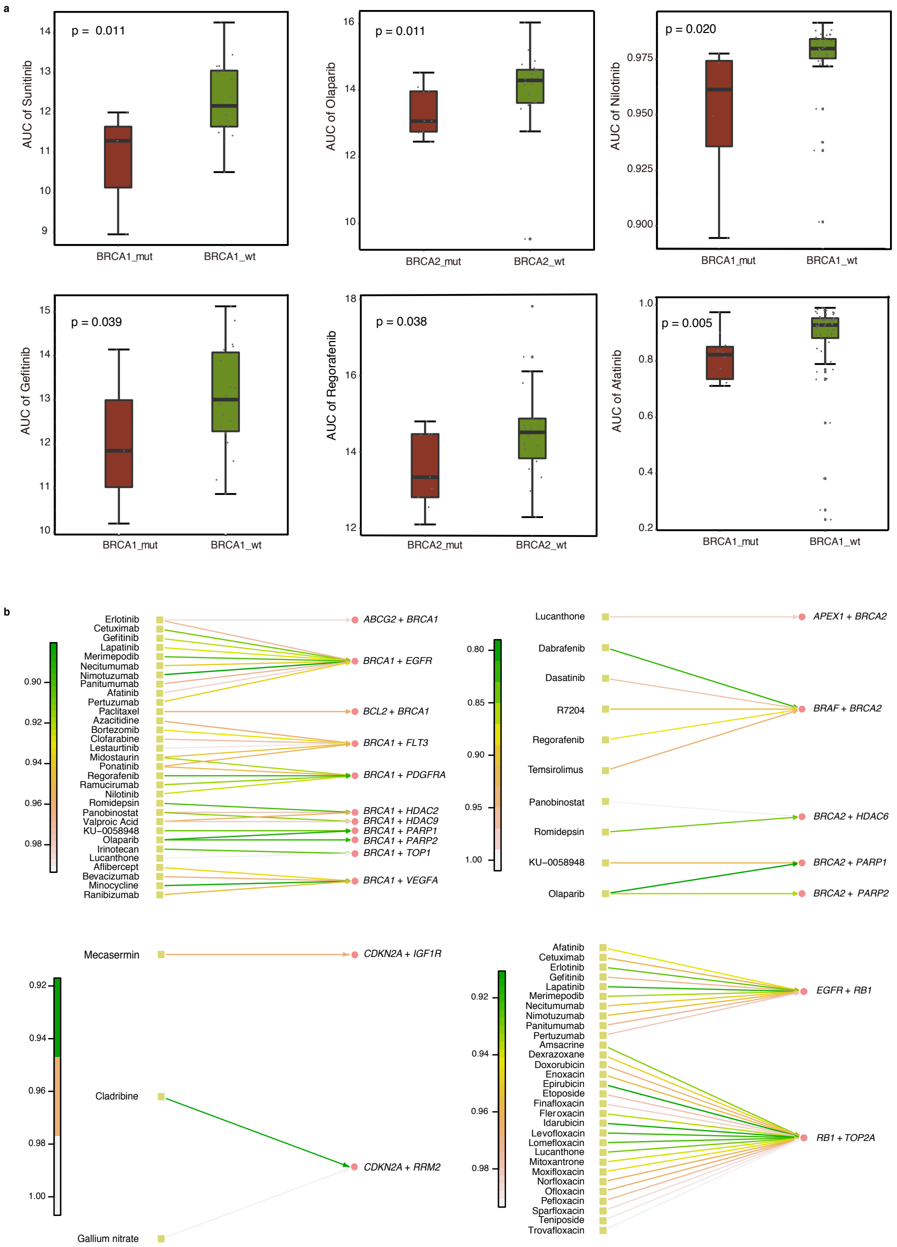

Supplementary Fig. 1. Boxplot and Bipartite.

a, Boxplot of one-sided Wilcoxon rank sum test based on SL (AUC). Box plots indicate median (middle line), 25th, 75th percentile (box) and 5th and 95th percentile (whiskers) as well as outliers (single points), n = 42 independent experiments in AUC of Sunitinib, n = 31 independent experiments in AUC of Olaparib, n = 40 independent experiments in AUC of Nilotinib, n = 84 independent experiments in AUC of Gefitinib, n = 27 independent experiments in AUC of Regorafenib, n = 64 independent experiments in AUC of Afatinib; Dots represent cancer cell lines. b, Bipartite graph between drug (yellow square) and SL pairs (pink circle). Side annotation bar represents DrugScore.

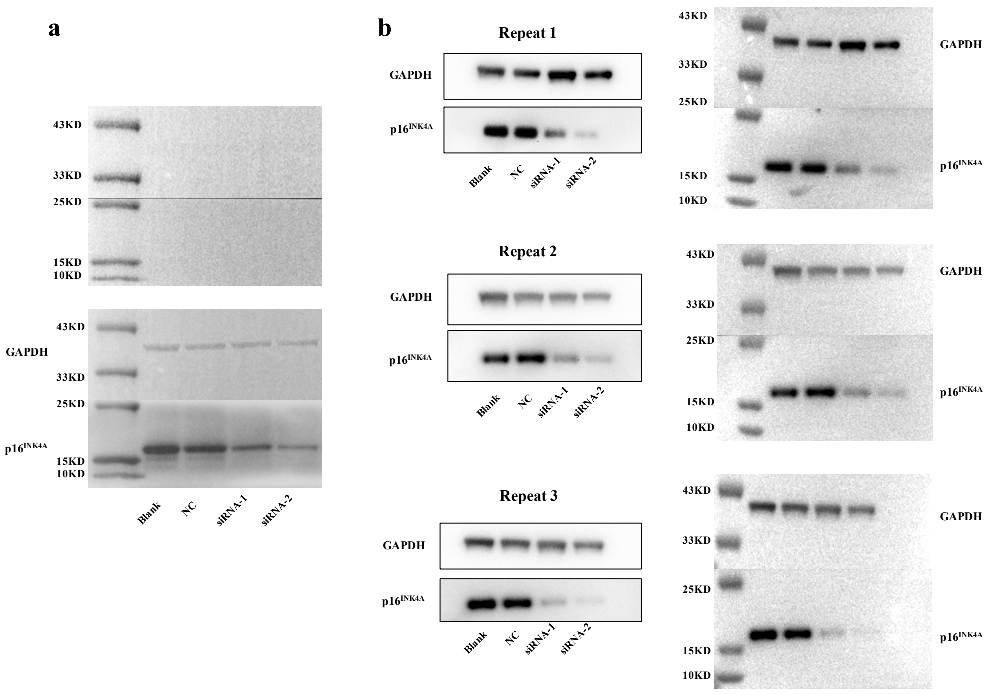

Supplementary Fig. 2. Knockdown efficiency of *CDKN2A* was determined by Western blot.

a. Full scans of figure 6(b).

b. Knockdown efficiency of *CDKN2A* was repeated at 3 times independently with similar results.

Supplementary Table 1: Supported drug repositioning results by literatures(Top 20)

Type	Rank	Drug	Known Indications	Repositioning of the Cancer	Target Gene	GeneA+GeneB	MutationGene	Score
SL	3	Rucaparib	Breast cancer	Hereditary Breast and Ovarian Cancer Syndrome	PARP1	BRCA1 + PARP1	BRCA1	0.913
SL	4	Rucaparib	Breast cancer	Hereditary Breast and Ovarian Cancer Syndrome	PARP1	BRCA2 + PARP1	BRCA2	0.894
SL	14	Romidepsin	Peripheral T-cell lymphoma	Hereditary Breast and Ovarian Cancer Syndrome	HDAC2	BRCA1 + HDAC2	BRCA1	0.648
SL	17	Erlotinib	Colon cancer; Glioma; Breast cancer; Head and neck cancer	Retinoblastoma	EGFR	EGFR + RB1	RB1	0.607
SL	18	Erlotinib	Pancreatic cancer	Hereditary Breast and Ovarian Cancer Syndrome	EGFR	BRCA1 + EGFR	BRCA1	0.583
SL	20	Rucaparib	Ovarian cancer	Hereditary Breast and Ovarian Cancer Syndrome	PARP2	BRCA2 + PARP2	BRCA2	0.564
SDL	6	Vandetanib	Solid tumours	Carcinoma, Pancreatic Ductal	EGFR	EGFR + NOTCH1	NOTCH1	0.687
SDL	20	Lapatinib	Breast cancer	Carcinoma, Pancreatic Ductal	EGFR	EGFR + NOTCH1	NOTCH1	0.428

Literatures:

1 D. C. Pearre and K. S. Tewari, “Targeted treatment of advanced ovarian cancer: Spotlight on rucaparib,” *Ther. Clin. Risk Manag.* , vol. 14, pp. 2189–2201, 2018

2 D. Pchejetski, A. Alfraidi, K. Sacco, H. Alshaker, A. Muhammad, and L. Monzon, “Histone deacetylases as new therapy targets for platinum-resistant epithelial ovarian cancer,” *J. Cancer Res. Clin. Oncol.* , vol. 142, no. 8, pp. 1659–1671, 2016

3 Y. Shao et al., “Erlotinib has tumor inhibitory effect in human retinoblastoma cells,” Biomed. Pharmacother., vol. 85, pp. 479–485, 2017.

4 K. Nakai, M. C. Hung, and H. Yamaguchi, “A perspective on anti-EGFR therapies targeting triple-negative breast cancer,” Am. J. Cancer Res., vol. 6, no. 8, pp. 1609–1623, 2016.

5 A. Turk and K. B. Wisinski, “PARP Inhibition in BRCA-Mutant Breast Cancer,” Cancer, vol. 124, no. 12, p. 2498, 2018.

6 A. D. Singhi et al., “Real-Time Targeted Genome Profile Analysis of Pancreatic Ductal Adenocarcinomas Identifies Genetic Alterations That Might Be Targeted With Existing Drugs or Used as Biomarkers,” Gastroenterology, vol. 156, no. 8, pp. 2242-2253.e4, 2019.

7 N. Walsh et al., “EGFR and HER2 inhibition in pancreatic cancer,” Invest New Drugs, vol. 31, pp. 558–566, 2013.

Supplementary Table 2: Clinical trial records of the top repurposable drug candidates based on SL and SDL

Class	Drug	Repositioning of cancer	Clinical trial title
SL	Olaparib	Hereditary Breast and Ovarian Cancer Syndrome	Palbociclib With Cetuximab and IMRT for Locally Advanced Squamous Cell Carcinoma
SL	Cetuximab	Retinoblastoma	Study of Treatment for Patients With Cancer of the Eye -Retinoblastoma
SL	Gefitinib	Retinoblastoma	PErsonalized TREatment of High-risk MAmmary Cancer - the PETREMAC Trial
SL	Lapatinib	Retinoblastoma	Vincristine, Carboplatin, and Etoposide or Observation Only in Treating Patients Who Have Undergone Surgery for Newly Diagnosed Retinoblastoma
SL	Paclitaxel	Hereditary Breast and Ovarian Cancer Syndrome	Molecular Phenotype Changes and Personalized Treatment for CRPC
SL	Pertuzumab	Retinoblastoma	Molecular Phenotype Changes and Personalized Treatment for CRPC
SL	Temsirolimus	Hereditary Breast and Ovarian Cancer Syndrome	-PATROL - Non-interventional Study (NIS) to Collect Clinical and Patient Reported Outcome Data in an Olaparib Treated BRCAm+ PSR Ovarian Cancer Population
SL	Epirubicin	Retinoblastoma	Veliparib, Carboplatin, and Paclitaxel in Treating Patients With Advanced Solid Cancer
SL	Etoposide	Retinoblastoma	Anastrozole, Palbociclib, Trastuzumab and Pertuzumab in Her-positive, Her2-positive Metastatic Breast
SL	Doxorubicin	Retinoblastoma	Temsirolimus and Vinorelbine Ditartrate in Treating Patients With Unresectable or Metastatic Solid Tumors
SDL	Vandetanib	Lymphoma	Testing Afatinib as a Potential Targeted Treatment in Cancers With HER2 Genetic Changes (MATCH-Subprotocol B)
SDL	Afatinib	Lymphoma	Serial Measurements of Molecular and Architectural Responses to Therapy (SMMART) PRIME Trial
SDL	Cetuximab	Precursor T-Cell Lymphoblastic Leukemia-Lymphoma	Safety, Tolerability, Pharmacokinetics and Preliminary Efficacy of SCT200 in Patients With Advanced Solid Tumors
SDL	Afatinib	Precursor T-Cell Lymphoblastic Leukemia-Lymphoma	CD19 CAR-T Cells for Patients With Relapse and Refractory CD19+ B-ALL.
SDL	Panitumumab	Lymphoma	A Phase 1 Dose-Escalation Study in Advanced Solid Tumors, Lymphomas or Multiple Myeloma
SDL	Lidocaine	Lymphoma	Serial Measurements of Molecular and Architectural Responses to Therapy (SMMART) PRIME Trial
SDL	Cetuximab	Lymphoma	A Study of LY2875358 in Japanese Participants With Advanced Cancer
SDL	Erlotinib	Lymphoma	S0528 Lapatinib and Everolimus in Treating Patients With Advanced Solid Tumors or Non-Hodgkin's Lymphoma
SDL	Erlotinib	Precursor T-Cell Lymphoblastic Leukemia-Lymphoma	Recalcitrant Pruritus in Cutaneous T-Cell Lymphoma
SDL	Gefitinib	Lymphoma	REAL3 Trial of Efficacy of EOX With/Without Panitumumab in Previously Untreated Adv OG Cancer
SDL	Lapatinib	Lymphoma	ted Therapy Directed by Genetic Testing in Treating Patients With Advanced Refractory Solid Tumors, Lymphomas, or Multiple Myeloma (The MATCH Screening Trial)
SDL	Pertuzumab	Lymphoma	Serial Measurements of Molecular and Architectural Responses to Therapy (SMMART) PRIME Trial
SDL	Vandetanib	Leukemia	Vandetanib and Bevacizumab in Treating Patients With Advanced Solid Tumors or Lymphoma
SDL	Pertuzumab	Precursor T-Cell Lymphoblastic Leukemia-Lymphoma	Vandetanib and Bevacizumab in Treating Patients With Advanced Solid Tumors or Lymphoma

å

Supplementary Table 3: Best repurposable drug candidates of SL and SDL

DrugName	Known indications	Repositioning of the cancer	TargetGene	Target_GeneID	GeneA+GeneB	MutationG	Mutation_GeneID	URL	RRA_score	Type
Lestaurtinib	Acute myeloid leuker	Hereditary Breast and Ovarian Cancer Syndrome	FLT3	2322	BRCA1+FLT3	BRCA1	672	http://gepia.cancer-pku.cn/detail.php?gene=BRCA1	0.0002524	SL
Olaparib	Ovarian Cancer	Hereditary Breast and Ovarian Cancer Syndrome	PARP2	10038	BRCA1+PARP2	BRCA1	672	http://gepia.cancer-pku.cn/detail.php?gene=BRCA1	0.001586	SL
Vemurafenib	Melanoma	Hereditary Breast and Ovarian Cancer Syndrome	BRAF	673	BRAF+BRCA2	BRCA2	675	http://gepia.cancer-pku.cn/detail.php?gene=BRCA2	0.0024652	SL
Fleroxacin	Bacterial infection	Retinoblastoma	TOP2A	7153	RB1+TOP2A	RB1	5925	http://gepia.cancer-pku.cn/detail.php?gene=RB1	0.0029413	SL
Rucaparib	Breast cancer	Hereditary Breast and Ovarian Cancer Syndrome	PARP2	10038	BRCA2+PARP2	BRCA2	675	http://gepia.cancer-pku.cn/detail.php?gene=BRCA2	0.0044669	SL
Gallium nitrate	Hypercalcemia	Melanoma-Pancreatic Cancer Syndrome	RRM2	6241	CDKN2A+RRM2	CDKN2A	1029	http://gepia.cancer-pku.cn/detail.php?gene=CDKN2A	0.0054857	SL
Afatinib	Non-small-cell lung c	Hereditary Breast and Ovarian Cancer Syndrome	EGFR	1956	BRCA1+EGFR	BRCA1	672	http://gepia.cancer-pku.cn/detail.php?gene=BRCA1	0.0063187	SL
Lomefloxacin	Bacterial infection	Retinoblastoma	TOP2A	7153	RB1+TOP2A	RB1	5925	http://gepia.cancer-pku.cn/detail.php?gene=RB1	0.0068045	SL
Cetuximab	Colorectal cancer	Retinoblastoma	EGFR	1956	EGFR+RB1	RB1	5925	http://gepia.cancer-pku.cn/detail.php?gene=RB1	0.0079804	SL
Bortezomib	NHL; MCL	Hereditary Breast and Ovarian Cancer Syndrome	FLT3	2322	BRCA1+FLT3	BRCA1	672	http://gepia.cancer-pku.cn/detail.php?gene=BRCA1	0.0089863	SL
Lucanthone	Cancer	Hereditary Breast and Ovarian Cancer Syndrome	APEX1	328	APEX1+BRCA2	BRCA2	675	http://gepia.cancer-pku.cn/detail.php?gene=BRCA2	0.010843	SL
Mecasermin	Growth failure	Multiple Endocrine Neoplasia;Type IV	IGF1R	3480	CDKN1B+IGF1R	CDKN1B	1027	http://gepia.cancer-pku.cn/detail.php?gene=CDKN1B	0.0121035	SL
Dabrafenib	Metastatic melanoma	Hereditary Breast and Ovarian Cancer Syndrome	BRAF	673	BRAF+BRCA2	BRCA2	675	http://gepia.cancer-pku.cn/detail.php?gene=BRCA2	0.0133356	SL
Panobinostat	Primary myelofibrosi	Hereditary Breast and Ovarian Cancer Syndrome	HDAC9	9734	BRCA1+HDAC9	BRCA1	672	http://gepia.cancer-pku.cn/detail.php?gene=BRCA1	0.0144409	SL
Valproic Acid	Bipolar disorder	Hereditary Breast and Ovarian Cancer Syndrome	HDAC2	3066	BRCA1+HDAC2	BRCA1	672	http://gepia.cancer-pku.cn/detail.php?gene=BRCA1	0.0156031	SL
Necitumumab	Squamous non-small	Retinoblastoma	EGFR	1956	EGFR+RB1	RB1	5925	http://gepia.cancer-pku.cn/detail.php?gene=RB1	0.0162103	SL
Enoxacin	Urinary tract infectior	Retinoblastoma	TOP2A	7153	RB1+TOP2A	RB1	5925	http://gepia.cancer-pku.cn/detail.php?gene=RB1	0.0172033	SL
Gefitinib	Urethral cancer; Blad	Retinoblastoma	EGFR	1956	EGFR+RB1	RB1	5925	http://gepia.cancer-pku.cn/detail.php?gene=RB1	0.0184211	SL
Panitumumab	Locally advanced hea	Retinoblastoma	EGFR	1956	EGFR+RB1	RB1	5925	http://gepia.cancer-pku.cn/detail.php?gene=RB1	0.020215	SL
Merimepodib	Refractory breast can	Retinoblastoma	EGFR	1956	EGFR+RB1	RB1	5925	http://gepia.cancer-pku.cn/detail.php?gene=RB1	0.0298069	SL
Dasatinib	Solid tumours; Multip	Hereditary Breast and Ovarian Cancer Syndrome	BRAF	673	BRAF+BRCA2	BRCA2	675	http://gepia.cancer-pku.cn/detail.php?gene=BRCA2	0.031015	SL
Dexrazoxane	Breast cancer	Retinoblastoma	TOP2A	7153	RB1+TOP2A	RB1	5925	http://gepia.cancer-pku.cn/detail.php?gene=RB1	0.033623	SL
Erlotinib	Colon cancer; Glioma	Hereditary Breast and Ovarian Cancer Syndrome	EGFR	1956	BRCA1+EGFR	BRCA1	672	http://gepia.cancer-pku.cn/detail.php?gene=BRCA1	0.0364663	SL
Panitumumab	Locally advanced hea	Hereditary Breast and Ovarian Cancer Syndrome	EGFR	1956	BRCA1+EGFR	BRCA1	672	http://gepia.cancer-pku.cn/detail.php?gene=BRCA1	0.0366513	SL
Mecasermin	IGF-1 deficiency	Multiple Endocrine Neoplasia;Type IV	IGF1R	3480	CDKN1B+IGF1R	CDKN1B	1027	http://gepia.cancer-pku.cn/detail.php?gene=CDKN1B	0.0373938	SL
Finafloxacin	Urinary tract infectior	Retinoblastoma	TOP2A	7153	RB1+TOP2A	RB1	5925	http://gepia.cancer-pku.cn/detail.php?gene=RB1	0.0394138	SL
Rucaparib	Ovarian cancer	Hereditary Breast and Ovarian Cancer Syndrome	PARP2	10038	BRCA1+PARP2	BRCA1	672	http://gepia.cancer-pku.cn/detail.php?gene=BRCA1	0.0447193	SL
Erlotinib	Pancreatic cancer	Retinoblastoma	EGFR	1956	EGFR+RB1	RB1	5925	http://gepia.cancer-pku.cn/detail.php?gene=RB1	0.0449236	SL
Vandetanib	Solid tumours	Retinoblastoma	EGFR	1956	EGFR+RB1	RB1	5925	http://gepia.cancer-pku.cn/detail.php?gene=RB1	0.0451489	SL
Trovafloxacin	Bacterial infection	Retinoblastoma	TOP2A	7153	RB1+TOP2A	RB1	5925	http://gepia.cancer-pku.cn/detail.php?gene=RB1	0.0488104	SL
Rucaparib	Ovarian cancer	Hereditary Breast and Ovarian Cancer Syndrome	PARP1	142	BRCA2+PARP1	BRCA2	675	http://gepia.cancer-pku.cn/detail.php?gene=BRCA2	0.048837	SL
Aflibercept	Ovarian cancer	Hereditary Breast and Ovarian Cancer Syndrome	VEGFA	7422	BRCA1+VEGFA	BRCA1	672	http://gepia.cancer-pku.cn/detail.php?gene=BRCA1	0.0517085	SL
Lestaurtinib	Acute myeloid leuker	Hereditary Breast and Ovarian Cancer Syndrome	FLT3	2322	BRCA1+FLT3	BRCA1	672	http://gepia.cancer-pku.cn/detail.php?gene=BRCA1	0.0529105	SL
Erlotinib	Non-small-cell lung c	Retinoblastoma	EGFR	1956	EGFR+RB1	RB1	5925	http://gepia.cancer-pku.cn/detail.php?gene=RB1	0.0550502	SL
Ponatinib	Acute lymphoblastic	Hereditary Breast and Ovarian Cancer Syndrome	FLT3	2322	BRCA1+FLT3	BRCA1	672	http://gepia.cancer-pku.cn/detail.php?gene=BRCA1	0.0557449	SL
Vorinostat	Cutaneous T-cell lym	Hereditary Breast and Ovarian Cancer Syndrome	HDAC6	10013	BRCA2+HDAC6	BRCA2	675	http://gepia.cancer-pku.cn/detail.php?gene=BRCA2	0.056496	SL
Romidepsin	Cutaneous T-cell lym	Hereditary Breast and Ovarian Cancer Syndrome	HDAC6	10013	BRCA2+HDAC6	BRCA2	675	http://gepia.cancer-pku.cn/detail.php?gene=BRCA2	0.0568319	SL
Panobinostat	Type 2 diabetes	Hereditary Breast and Ovarian Cancer Syndrome	HDAC2	3066	BRCA1+HDAC2	BRCA1	672	http://gepia.cancer-pku.cn/detail.php?gene=BRCA1	0.0590688	SL
Erlotinib	Pancreatic cancer	Hereditary Breast and Ovarian Cancer Syndrome	ABCG2	9429	ABCG2+BRCA1	BRCA1	672	http://gepia.cancer-pku.cn/detail.php?gene=BRCA1	0.0669754	SL
Lapatinib	Breast cancer	Retinoblastoma	EGFR	1956	EGFR+RB1	RB1	5925	http://gepia.cancer-pku.cn/detail.php?gene=RB1	0.0701602	SL
Valproic Acid	Bipolar disorder	Hereditary Breast and Ovarian Cancer Syndrome	HDAC9	9734	BRCA1+HDAC9	BRCA1	672	http://gepia.cancer-pku.cn/detail.php?gene=BRCA1	0.0724235	SL
Sunitinib	Advanced renal cell c	Hereditary Breast and Ovarian Cancer Syndrome	PDGFRA	5156	BRCA1+PDGFRA	BRCA1	672	http://gepia.cancer-pku.cn/detail.php?gene=BRCA1	0.077946	SL
Panobinostat	Type 2 diabetes	Hereditary Breast and Ovarian Cancer Syndrome	HDAC9	9734	BRCA1+HDAC9	BRCA1	672	http://gepia.cancer-pku.cn/detail.php?gene=BRCA1	0.0864597	SL
Romidepsin	Peripheral T-cell lym	Hereditary Breast and Ovarian Cancer Syndrome	HDAC6	10013	BRCA2+HDAC6	BRCA2	675	http://gepia.cancer-pku.cn/detail.php?gene=BRCA2	0.0867905	SL
Paclitaxel	Cancer	Hereditary Breast and Ovarian Cancer Syndrome	BCL2	596	BCL2+BRCA1	BRCA1	672	http://gepia.cancer-pku.cn/detail.php?gene=BRCA1	0.0868356	SL
Pertuzumab	Breast cancer	Retinoblastoma	EGFR	1956	EGFR+RB1	RB1	5925	http://gepia.cancer-pku.cn/detail.php?gene=RB1	0.08741	SL
Aflibercept	Metastatic colorectal	Hereditary Breast and Ovarian Cancer Syndrome	VEGFA	7422	BRCA1+VEGFA	BRCA1	672	http://gepia.cancer-pku.cn/detail.php?gene=BRCA1	0.0901918	SL
Lucanthone	Cancer	Lymphangioleiomyomatosis	TOP1	7150	TOP1+TSC1	TSC1	7248	http://gepia.cancer-pku.cn/detail.php?gene=TSC1	0.0905208	SL
Norfloxacin	Bacterial infection	Retinoblastoma	TOP2A	7153	RB1+TOP2A	RB1	5925	http://gepia.cancer-pku.cn/detail.php?gene=RB1	0.0969732	SL
Sunitinib	Advanced renal cell c	Hereditary Breast and Ovarian Cancer Syndrome	FLT3	2322	BRCA1+FLT3	BRCA1	672	http://gepia.cancer-pku.cn/detail.php?gene=BRCA1	0.0973652	SL
Teniposide	Acute lymphoblastic	Retinoblastoma	TOP2A	7153	RB1+TOP2A	RB1	5925	http://gepia.cancer-pku.cn/detail.php?gene=RB1	0.0976813	SL
Dabrafenib	Melanoma	Hereditary Breast and Ovarian Cancer Syndrome	BRAF	673	BRAF+BRCA2	BRCA2	675	http://gepia.cancer-pku.cn/detail.php?gene=BRCA2	0.099806	SL
Panobinostat	Primary myelofibrosi	Hereditary Breast and Ovarian Cancer Syndrome	HDAC2	3066	BRCA1+HDAC2	BRCA1	672	http://gepia.cancer-pku.cn/detail.php?gene=BRCA1	0.1001151	SL
Merimepodib	Head and neck squar	Retinoblastoma	EGFR	1956	EGFR+RB1	RB1	5925	http://gepia.cancer-pku.cn/detail.php?gene=RB1	0.1023235	SL
Necitumumab	Colorectal cancer	Hereditary Breast and Ovarian Cancer Syndrome	EGFR	1956	BRCA1+EGFR	BRCA1	672	http://gepia.cancer-pku.cn/detail.php?gene=BRCA1	0.1039433	SL
Temsirolimus	Renal cell carcinoma	Hereditary Breast and Ovarian Cancer Syndrome	BRAF	673	BRAF+BRCA2	BRCA2	675	http://gepia.cancer-pku.cn/detail.php?gene=BRCA2	0.1055108	SL
Vorinostat	Cutaneous T-cell lym	Hereditary Breast and Ovarian Cancer Syndrome	HDAC9	9734	BRCA1+HDAC9	BRCA1	672	http://gepia.cancer-pku.cn/detail.php?gene=BRCA1	0.1059155	SL
Cetuximab	Colorectal cancer	Hereditary Breast and Ovarian Cancer Syndrome	EGFR	1956	BRCA1+EGFR	BRCA1	672	http://gepia.cancer-pku.cn/detail.php?gene=BRCA1	0.108545	SL
Necitumumab	Colorectal cancer	Retinoblastoma	EGFR	1956	EGFR+RB1	RB1	5925	http://gepia.cancer-pku.cn/detail.php?gene=RB1	0.1128717	SL
Motexafin gadoliniur	Human immunodefici	Melanoma-Pancreatic Cancer Syndrome	RRM2	6241	CDKN2A+RRM2	CDKN2A	1029	http://gepia.cancer-pku.cn/detail.php?gene=CDKN2A	0.1144958	SL
Mecasermin	Growth failure	Melanoma-Pancreatic Cancer Syndrome	IGF1R	3480	CDKN2A+IGF1R	CDKN2A	1029	http://gepia.cancer-pku.cn/detail.php?gene=CDKN2A	0.1197175	SL
Clofarabine	Acute lymphoblastic	Hereditary Breast and Ovarian Cancer Syndrome	FLT3	2322	BRCA1+FLT3	BRCA1	672	http://gepia.cancer-pku.cn/detail.php?gene=BRCA1	0.1212957	SL
Nimotuzumab	Head and neck cancer	Hereditary Breast and Ovarian Cancer Syndrome	EGFR	1956	BRCA1+EGFR	BRCA1	672	http://gepia.cancer-pku.cn/detail.php?gene=BRCA1	0.1235952	SL
KU-0058948	Ovarian cancer	Hereditary Breast and Ovarian Cancer Syndrome	PARP1	142	BRCA2+PARP1	BRCA2	675	http://gepia.cancer-pku.cn/detail.php?gene=BRCA2	0.125241	SL
Clofarabine	Myelodysplastic synd	Hereditary Breast and Ovarian Cancer Syndrome	FLT3	2322	BRCA1+FLT3	BRCA1	672	http://gepia.cancer-pku.cn/detail.php?gene=BRCA1	0.1271165	SL
Midostaurin	Colon cancer; Breast	Hereditary Breast and Ovarian Cancer Syndrome	FLT3	2322	BRCA1+FLT3	BRCA1	672	http://gepia.cancer-pku.cn/detail.php?gene=BRCA1	0.1376365	SL
Moxifloxacin	Bacterial infection	Retinoblastoma	TOP2A	7153	RB1+TOP2A	RB1	5925	http://gepia.cancer-pku.cn/detail.php?gene=RB1	0.1416358	SL
Topotecan	Ovarian cancer	Hereditary Breast and Ovarian Cancer Syndrome	TOP1	7150	BRCA1+TOP1	BRCA1	672	http://gepia.cancer-pku.cn/detail.php?gene=BRCA1	0.1572623	SL
Ponatinib	Acute lymphoblastic	Hereditary Breast and Ovarian Cancer Syndrome	PDGFRA	5156	BRCA1+PDGFRA	BRCA1	672	http://gepia.cancer-pku.cn/detail.php?gene=BRCA1	0.1588496	SL
Romidepsin	Renal cell carcinoma;	Hereditary Breast and Ovarian Cancer Syndrome	HDAC6	10013	BRCA2+HDAC6	BRCA2	675	http://gepia.cancer-pku.cn/detail.php?gene=BRCA2	0.1601788	SL
Gefitinib	Urethral cancer; Blad	Hereditary Breast and Ovarian Cancer Syndrome	EGFR	1956	BRCA1+EGFR	BRCA1	672	http://gepia.cancer-pku.cn/detail.php?gene=BRCA1	0.1633908	SL
Lapatinib	Refractory metastatic	Hereditary Breast and Ovarian Cancer Syndrome	EGFR	1956	BRCA1+EGFR	BRCA1	672	http://gepia.cancer-pku.cn/detail.php?gene=BRCA1	0.1655432	SL
Rucaparib	Breast cancer	Hereditary Breast and Ovarian Cancer Syndrome	PARP2	10038	BRCA1+PARP2	BRCA1	672	http://gepia.cancer-pku.cn/detail.php?gene=BRCA1	0.171404	SL
Olaparib	Ovarian Cancer	Hereditary Breast and Ovarian Cancer Syndrome	PARP1	142	BRCA2+PARP1	BRCA2	675	http://gepia.cancer-pku.cn/detail.php?gene=BRCA2	0.1743815	SL
Valproic Acid	Epilepsy	Hereditary Breast and Ovarian Cancer Syndrome	HDAC2	3066	BRCA1+HDAC2	BRCA1	672	http://gepia.cancer-pku.cn/detail.php?gene=BRCA1	0.1781746	SL
Lucanthone	Cancer	Retinoblastoma	TOP2A	7153	RB1+TOP2A	RB1	5925	http://gepia.cancer-pku.cn/detail.php?gene=RB1	0.1809003	SL
Rucaparib	Ovarian cancer	Hereditary Breast and Ovarian Cancer Syndrome	PARP1	142	BRCA1+PARP1	BRCA1	672	http://gepia.cancer-pku.cn/detail.php?gene=BRCA1	0.1813638	SL
Vandetanib	Solid tumours	Hereditary Breast and Ovarian Cancer Syndrome	EGFR	1956	BRCA1+EGFR	BRCA1	672	http://gepia.cancer-pku.cn/detail.php?gene=BRCA1	0.183196	SL
Merimepodib	Head and neck squar	Hereditary Breast and Ovarian Cancer Syndrome	EGFR	1956	BRCA1+EGFR	BRCA1	672	http://gepia.cancer-pku.cn/detail.php?gene=BRCA1	0.1840658	SL
Vorinostat	Cutaneous T-cell lym	Hereditary Breast and Ovarian Cancer Syndrome	HDAC2	3066	BRCA1+HDAC2	BRCA1	672	http://gepia.cancer-pku.cn/detail.php?gene=BRCA1	0.1932522	SL
Necitumumab	Squamous non-small	Hereditary Breast and Ovarian Cancer Syndrome	EGFR	1956	BRCA1+EGFR	BRCA1	672	http://gepia.cancer-pku.cn/detail.php?gene=BRCA1	0.1968151	SL
Nimotuzumab	Head and neck cancer	Retinoblastoma	EGFR	1956	EGFR+RB1	RB1	5925	http://gepia.cancer-pku.cn/detail.php?gene=RB1	0.1977975	SL
Topotecan	Ovarian cancer	Lymphangioleiomyomatosis	TOP1	7150	TOP1+TSC1	TSC1	7248	http://gepia.cancer-pku.cn/detail.php?gene=TSC1	0.2004788	SL
Vemurafenib	Metastatic melanoma	Hereditary Breast and Ovarian Cancer Syndrome	BRAF	673	BRAF+BRCA2	BRCA2	675	http://gepia.cancer-pku.cn/detail.php?gene=BRCA2	0.205277	SL
Necitumumab	Non-small-cell lung c	Retinoblastoma	EGFR	1956	EGFR+RB1	RB1	5925	http://gepia.cancer-pku.cn/detail.php?gene=RB1	0.2070934	SL
Idarubicin	Acute myeloid leuker	Retinoblastoma	TOP2A	7153	RB1+TOP2A	RB1	5925	http://gepia.cancer-pku.cn/detail.php?gene=RB1	0.2197926	SL
Vemurafenib	Thyroid cancer	Hereditary Breast and Ovarian Cancer Syndrome	BRAF	673	BRAF+BRCA2	BRCA2	675	http://gepia.cancer-pku.cn/detail.php?gene=BRCA2	0.2279056	SL
Olaparib	Ovarian Cancer	Hereditary Breast and Ovarian Cancer Syndrome	PARP1	142	BRCA1+PARP1	BRCA1	672	http://gepia.cancer-pku.cn/detail.php?gene=BRCA1	0.2344338	SL
Enoxacin	Bacterial infection	Retinoblastoma	TOP2A	7153	RB1+TOP2A	RB1	5925	http://gepia.cancer-pku.cn/detail.php?gene=RB1	0.2361583	SL
Panitumumab	Colorectal cancer	Retinoblastoma	EGFR	1956	EGFR+RB1	RB1	5925	http://gepia.cancer-pku.cn/detail.php?gene=RB1	0.2390349	SL
Midostaurin	Breast cancer; Colore	Hereditary Breast and Ovarian Cancer Syndrome	PDGFRA	5156	BRCA1+PDGFRA	BRCA1	672	http://gepia.cancer-pku.cn/detail.php?gene=BRCA1	0.2476437	SL
Amsacrine	Acute lymphoblastic	Retinoblastoma	TOP2A	7153	RB1+TOP2A	RB1	5925	http://gepia.cancer-pku.cn/detail.php?gene=RB1	0.2506212	SL
Regorafenib	Metastatic colorectal	Hereditary Breast and Ovarian Cancer Syndrome	BRAF	673	BRAF+BRCA2	BRCA2	675	http://gepia.cancer-pku.cn/detail.php?gene=BRCA2	0.2508768	SL
Epirubicin	Cancers	Retinoblastoma	TOP2A	7153	RB1+TOP2A	RB1	5925	http://gepia.cancer-pku.cn/detail.php?gene=RB1	0.2610686	SL
Minocycline	Bacterial infection	Hereditary Breast and Ovarian Cancer Syndrome	VEGFA	7422	BRCA1+VEGFA	BRCA1	672	http://gepia.cancer-pku.cn/detail.php?gene=BRCA1	0.2655433	SL
Merimepodib	Gastric cancer	Retinoblastoma	EGFR	1956	EGFR+RB1	RB1	5925	http://gepia.cancer-pku.cn/detail.php?gene=RB1	0.2795967	SL
Gefitinib	Cancers	Retinoblastoma	EGFR	1956	EGFR+RB1	RB1	5925	http://gepia.cancer-pku.cn/detail.php?gene=RB1	0.2797344	SL
Gefitinib	Head and neck cancer	Hereditary Breast and Ovarian Cancer Syndrome	EGFR	1956	BRCA1+EGFR	BRCA1	672	http://gepia.cancer-pku.cn/detail.php?gene=BRCA1	0.2876208	SL
Gefitinib	Cancers	Hereditary Breast and Ovarian Cancer Syndrome	EGFR	1956	BRCA1+EGFR	BRCA1	672	http://gepia.cancer-pku.cn/detail.php?gene=BRCA1	0.2963429	SL
Midostaurin	Breast cancer; Colore	Hereditary Breast and Ovarian Cancer Syndrome	FLT3	2322	BRCA1+FLT3	BRCA1	672	http://gepia.cancer-pku.cn/detail.php?gene=BRCA1	0.2971899	SL
Azacitidine	Myelodysplastic synd	Hereditary Breast and Ovarian Cancer Syndrome	FLT3	2322	BRCA1+FLT3	BRCA1	672	http://gepia.cancer-pku.cn/detail.php?gene=BRCA1	0.2992243	SL
Erlotinib	Non-small-cell lung c	Hereditary Breast and Ovarian Cancer Syndrome	EGFR	1956	BRCA1+EGFR	BRCA1	672	http://gepia.cancer-pku.cn/detail.php?gene=BRCA1	0.3049914	SL
Dasatinib	Chronic myelogenous	Hereditary Breast and Ovarian Cancer Syndrome	BRAF	673	BRAF+BRCA2	BRCA2	675	http://gepia.cancer-pku.cn/detail.php?gene=BRCA2	0.3060758	SL
Motexafin gadoliniur	Brain cancer	Melanoma-Pancreatic Cancer Syndrome	RRM2	6241	CDKN2A+RRM2	CDKN2A	1029	http://gepia.cancer-pku.cn/detail.php?gene=CDKN2A	0.3097754	SL
Nilotinib	Chronic myelogenous	Hereditary Breast and Ovarian Cancer Syndrome	PDGFRA	5156	BRCA1+PDGFRA	BRCA1	672	http://gepia.cancer-pku.cn/detail.php?gene=BRCA1	0.3142372	SL
Midostaurin	Colon cancer; Breast	Hereditary Breast and Ovarian Cancer Syndrome	PDGFRA	5156	BRCA1+PDGFRA	BRCA1	672	http://gepia.cancer-pku.cn/detail.php?gene=BRCA1	0.3225753	SL
Olaparib	Ovarian Cancer	Hereditary Breast and Ovarian Cancer Syndrome	PARP2	10038	BRCA2+PARP2	BRCA2	675	http://gepia.cancer-pku.cn/detail.php?gene=BRCA2	0.3281023	SL
Necitumumab	Non-small-cell lung c	Hereditary Breast and Ovarian Cancer Syndrome	EGFR	1956	BRCA1+EGFR	BRCA1	672	http://gepia.cancer-pku.cn/detail.php?gene=BRCA1	0.329717	SL
Irinotecan	Colorectal cancer	Lymphangioleiomyomatosis	TOP1	7150	TOP1+TSC1	TSC1	7248	http://gepia.cancer-pku.cn/detail.php?gene=TSC1	0.3305073	SL
Pefloxacin	Gonococcal urethritis	Retinoblastoma	TOP2A	7153	RB1+TOP2A	RB1	5925	http://gepia.cancer-pku.cn/detail.php?gene=RB1	0.3313194	SL
Bevacizumab	Glaucoma	Hereditary Breast and Ovarian Cancer Syndrome	VEGFA	7422	BRCA1+VEGFA	BRCA1	672	http://gepia.cancer-pku.cn/detail.php?gene=BRCA1	0.3445255	SL
Romidepsin	Renal cell carcinoma;	Hereditary Breast and Ovarian Cancer Syndrome	HDAC2	3066	BRCA1+HDAC2	BRCA1	672	http://gepia.cancer-pku.cn/detail.php?gene=BRCA1	0.3447546	SL
Afatinib	Non-small-cell lung c	Retinoblastoma	EGFR	1956	EGFR+RB1	RB1	5925	http://gepia.cancer-pku.cn/detail.php?gene=RB1	0.3533056	SL
Merimepodib	Gastric cancer	Hereditary Breast and Ovarian Cancer Syndrome	EGFR	1956	BRCA1+EGFR	BRCA1	672	http://gepia.cancer-pku.cn/detail.php?gene=BRCA1	0.3604332	SL
Etoposide	Cancers	Retinoblastoma	TOP2A	7153	RB1+TOP2A	RB1	5925	http://gepia.cancer-pku.cn/detail.php?gene=RB1	0.3676343	SL
Pertuzumab	Breast cancer	Hereditary Breast and Ovarian Cancer Syndrome	EGFR	1956	BRCA1+EGFR	BRCA1	672	http://gepia.cancer-pku.cn/detail.php?gene=BRCA1	0.3687895	SL
Irinotecan	Colorectal cancer	Hereditary Breast and Ovarian Cancer Syndrome	TOP1	7150	BRCA1+TOP1	BRCA1	672	http://gepia.cancer-pku.cn/detail.php?gene=BRCA1	0.3772331	SL
Lapatinib	Refractory metastatic	Retinoblastoma	EGFR	1956	EGFR+RB1	RB1	5925	http://gepia.cancer-pku.cn/detail.php?gene=RB1	0.395267	SL
Doxorubicin	Cancers	Retinoblastoma	TOP2A	7153	RB1+TOP2A	RB1	5925	http://gepia.cancer-pku.cn/detail.php?gene=RB1	0.4059567	SL
Ofloxacin	Bacterial infection	Retinoblastoma	TOP2A	7153	RB1+TOP2A	RB1	5925	http://gepia.cancer-pku.cn/detail.php?gene=RB1	0.4121771	SL
Romidepsin	Cutaneous T-cell lym	Hereditary Breast and Ovarian Cancer Syndrome	HDAC2	3066	BRCA1+HDAC2	BRCA1	672	http://gepia.cancer-pku.cn/detail.php?gene=BRCA1	0.4160209	SL
Lestaurtinib	Psoriasis	Hereditary Breast and Ovarian Cancer Syndrome	FLT3	2322	BRCA1+FLT3	BRCA1	672	http://gepia.cancer-pku.cn/detail.php?gene=BRCA1	0.4275699	SL
Erlotinib	Colon cancer; Glioma	Hereditary Breast and Ovarian Cancer Syndrome	ABCG2	9429	ABCG2+BRCA1	BRCA1	672	http://gepia.cancer-pku.cn/detail.php?gene=BRCA1	0.4434392	SL
Levofloxacin	Bacterial infection	Retinoblastoma	TOP2A	7153	RB1+TOP2A	RB1	5925	http://gepia.cancer-pku.cn/detail.php?gene=RB1	0.4482707	SL
Merimepodib	Refractory breast can	Hereditary Breast and Ovarian Cancer Syndrome	EGFR	1956	BRCA1+EGFR	BRCA1	672	http://gepia.cancer-pku.cn/detail.php?gene=BRCA1	0.4519335	SL
Lapatinib	Breast cancer	Hereditary Breast and Ovarian Cancer Syndrome	EGFR	1956	BRCA1+EGFR	BRCA1	672	http://gepia.cancer-pku.cn/detail.php?gene=BRCA1	0.4526073	SL
Panobinostat	Primary myelofibrosi	Hereditary Breast and Ovarian Cancer Syndrome	HDAC6	10013	BRCA2+HDAC6	BRCA2	675	http://gepia.cancer-pku.cn/detail.php?gene=BRCA2	0.4634527	SL
Erlotinib	Non-small-cell lung c	Hereditary Breast and Ovarian Cancer Syndrome	ABCG2	9429	ABCG2+BRCA1	BRCA1	672	http://gepia.cancer-pku.cn/detail.php?gene=BRCA1	0.4649498	SL
Gefitinib	Head and neck cancer	Retinoblastoma	EGFR	1956	EGFR+RB1	RB1	5925	http://gepia.cancer-pku.cn/detail.php?gene=RB1	0.4712972	SL
Bortezomib	Multiple myeloma	Hereditary Breast and Ovarian Cancer Syndrome	FLT3	2322	BRCA1+FLT3	BRCA1	672	http://gepia.cancer-pku.cn/detail.php?gene=BRCA1	0.4716216	SL
Mitoxantrone	Cancers	Retinoblastoma	TOP2A	7153	RB1+TOP2A	RB1	5925	http://gepia.cancer-pku.cn/detail.php?gene=RB1	0.472626	SL

Lucanthone	Cancer	Hereditary Breast and Ovarian Cancer Syndrome	TOP1	7150	BRCA1+TOP1	BRCA1	672	http://gepia.cancer-pku.cn/detail.php?gene=BRCA1	0.4777507	SL
Mecasermin	IGF-1 deficiency	Melanoma-Pancreatic Cancer Syndrome	IGF1R	3480	CDKN2A+IGF1R	CDKN2A	1029	http://gepia.cancer-pku.cn/detail.php?gene=CDKN2A	0.4831542	SL
Panobinostat	Type 2 diabetes	Hereditary Breast and Ovarian Cancer Syndrome	HDAC6	10013	BRCA2+HDAC6	BRCA2	675	http://gepia.cancer-pku.cn/detail.php?gene=BRCA2	0.5077461	SL
Sunitinib	Gastrointestinal cance	Hereditary Breast and Ovarian Cancer Syndrome	PDGFRA	5156	BRCA1+PDGFRA	BRCA1	672	http://gepia.cancer-pku.cn/detail.php?gene=BRCA1	0.5440494	SL
Rucaparib	Ovarian cancer	Hereditary Breast and Ovarian Cancer Syndrome	PARP2	10038	BRCA2+PARP2	BRCA2	675	http://gepia.cancer-pku.cn/detail.php?gene=BRCA2	0.5644088	SL
Ranibizumab	Hereditary hemorrhag	Hereditary Breast and Ovarian Cancer Syndrome	VEGFA	7422	BRCA1+VEGFA	BRCA1	672	http://gepia.cancer-pku.cn/detail.php?gene=BRCA1	0.5784458	SL
Erlotinib	Pancreatic cancer	Hereditary Breast and Ovarian Cancer Syndrome	EGFR	1956	BRCA1+EGFR	BRCA1	672	http://gepia.cancer-pku.cn/detail.php?gene=BRCA1	0.5830572	SL
Erlotinib	Colon cancer; Glioma	Retinoblastoma	EGFR	1956	EGFR+RB1	RB1	5925	http://gepia.cancer-pku.cn/detail.php?gene=RB1	0.6067572	SL
KU-0058948	Ovarian cancer	Hereditary Breast and Ovarian Cancer Syndrome	PARP1	142	BRCA1+PARP1	BRCA1	672	http://gepia.cancer-pku.cn/detail.php?gene=BRCA1	0.6095027	SL
Cladribine	Hairy cell leukemia	Melanoma-Pancreatic Cancer Syndrome	RRM2	6241	CDKN2A+RRM2	CDKN2A	1029	http://gepia.cancer-pku.cn/detail.php?gene=CDKN2A	0.6460937	SL
Romidepsin	Peripheral T-cell lymp	Hereditary Breast and Ovarian Cancer Syndrome	HDAC2	3066	BRCA1+HDAC2	BRCA1	672	http://gepia.cancer-pku.cn/detail.php?gene=BRCA1	0.6475432	SL
Sunitinib	Gastrointestinal cance	Hereditary Breast and Ovarian Cancer Syndrome	FLT3	2322	BRCA1+FLT3	BRCA1	672	http://gepia.cancer-pku.cn/detail.php?gene=BRCA1	0.6516035	SL
Lestaurtinib	Myeloid leukemia	Hereditary Breast and Ovarian Cancer Syndrome	FLT3	2322	BRCA1+FLT3	BRCA1	672	http://gepia.cancer-pku.cn/detail.php?gene=BRCA1	0.6772709	SL
Valrubicin	Bladder cancer	Retinoblastoma	TOP2A	7153	RB1+TOP2A	RB1	5925	http://gepia.cancer-pku.cn/detail.php?gene=RB1	0.6939272	SL
Valrubicin	Psoriasis	Retinoblastoma	TOP2A	7153	RB1+TOP2A	RB1	5925	http://gepia.cancer-pku.cn/detail.php?gene=RB1	0.7045685	SL
Vandetanib	Solid tumours	Hereditary Breast and Ovarian Cancer Syndrome	VEGFA	7422	BRCA1+VEGFA	BRCA1	672	http://gepia.cancer-pku.cn/detail.php?gene=BRCA1	0.7217885	SL
Sparfloxacin	Bacterial infection	Retinoblastoma	TOP2A	7153	RB1+TOP2A	RB1	5925	http://gepia.cancer-pku.cn/detail.php?gene=RB1	0.7750727	SL
R7204	Metastatic melanoma	Hereditary Breast and Ovarian Cancer Syndrome	BRAF	673	BRAF+BRCA2	BRCA2	675	http://gepia.cancer-pku.cn/detail.php?gene=BRCA2	0.8338741	SL
Valproic Acid	Epilepsy	Hereditary Breast and Ovarian Cancer Syndrome	HDAC9	9734	BRCA1+HDAC9	BRCA1	672	http://gepia.cancer-pku.cn/detail.php?gene=BRCA1	0.8664848	SL
Ramucirumab	Gastric cancer	Hereditary Breast and Ovarian Cancer Syndrome	PDGFRA	5156	BRCA1+PDGFRA	BRCA1	672	http://gepia.cancer-pku.cn/detail.php?gene=BRCA1	0.8813722	SL
Rucaparib	Breast cancer	Hereditary Breast and Ovarian Cancer Syndrome	PARP1	142	BRCA2+PARP1	BRCA2	675	http://gepia.cancer-pku.cn/detail.php?gene=BRCA2	0.8939161	SL
Rucaparib	Breast cancer	Hereditary Breast and Ovarian Cancer Syndrome	PARP1	142	BRCA1+PARP1	BRCA1	672	http://gepia.cancer-pku.cn/detail.php?gene=BRCA1	0.9126746	SL
Regorafenib	Metastatic colorectal	Hereditary Breast and Ovarian Cancer Syndrome	PDGFRA	5156	BRCA1+PDGFRA	BRCA1	672	http://gepia.cancer-pku.cn/detail.php?gene=BRCA1	0.916223	SL
Panitumumab	Colorectal cancer	Hereditary Breast and Ovarian Cancer Syndrome	EGFR	1956	BRCA1+EGFR	BRCA1	672	http://gepia.cancer-pku.cn/detail.php?gene=BRCA1	0.9468674	SL
Necitumumab	Non-small-cell lung ca	Carcinoma, Pancreatic Ductal	EGFR	1956	EGFR+NOTCH1	NOTCH1	4851	http://gepia.cancer-pku.cn/detail.php?gene=NOTCH1	0.0013177	SDL
Afatinib	Non-small-cell lung ca	Leukemia, T-Cell	EGFR	1956	EGFR+NOTCH1	NOTCH1	4851	http://gepia.cancer-pku.cn/detail.php?gene=NOTCH1	0.0047867	SDL
Vandetanib	Solid tumours	Lymphoma	EGFR	1956	EGFR+NOTCH1	NOTCH1	4851	http://gepia.cancer-pku.cn/detail.php?gene=NOTCH1	0.0073987	SDL
Gefitinib	Cancers	Carcinoma, Pancreatic Ductal	EGFR	1956	EGFR+NOTCH1	NOTCH1	4851	http://gepia.cancer-pku.cn/detail.php?gene=NOTCH1	0.0075909	SDL
Erlotinib	Colon cancer; Glioma	Carcinoma, Pancreatic Ductal	EGFR	1956	EGFR+NOTCH1	NOTCH1	4851	http://gepia.cancer-pku.cn/detail.php?gene=NOTCH1	0.0077142	SDL
Cetuximab	Colorectal cancer	Leukemia, T-Cell	EGFR	1956	EGFR+NOTCH1	NOTCH1	4851	http://gepia.cancer-pku.cn/detail.php?gene=NOTCH1	0.0135369	SDL
Necitumumab	Colorectal cancer	Lymphoma	EGFR	1956	EGFR+NOTCH1	NOTCH1	4851	http://gepia.cancer-pku.cn/detail.php?gene=NOTCH1	0.0152376	SDL
Erlotinib	Colon cancer; Glioma	Leukemia, T-Cell	EGFR	1956	EGFR+NOTCH1	NOTCH1	4851	http://gepia.cancer-pku.cn/detail.php?gene=NOTCH1	0.0161195	SDL
Lidocaine	Anesthesia	Carcinoma, Pancreatic Ductal	EGFR	1956	EGFR+NOTCH1	NOTCH1	4851	http://gepia.cancer-pku.cn/detail.php?gene=NOTCH1	0.0175679	SDL
Necitumumab	Non-small-cell lung ca	Leukemia, T-Cell	EGFR	1956	EGFR+NOTCH1	NOTCH1	4851	http://gepia.cancer-pku.cn/detail.php?gene=NOTCH1	0.0212555	SDL
Lidocaine	Anesthesia	Leukemia, T-Cell	EGFR	1956	EGFR+NOTCH1	NOTCH1	4851	http://gepia.cancer-pku.cn/detail.php?gene=NOTCH1	0.027715	SDL
Afatinib	Non-small-cell lung ca	Lymphoma	EGFR	1956	EGFR+NOTCH1	NOTCH1	4851	http://gepia.cancer-pku.cn/detail.php?gene=NOTCH1	0.0296036	SDL
Cetuximab	Colorectal cancer	Precursor T-Cell Lymphoblastic Leukemia-Lymphoma	EGFR	1956	EGFR+NOTCH1	NOTCH1	4851	http://gepia.cancer-pku.cn/detail.php?gene=NOTCH1	0.0359956	SDL
Necitumumab	Non-small-cell lung ca	Precursor T-Cell Lymphoblastic Leukemia-Lymphoma	EGFR	1956	EGFR+NOTCH1	NOTCH1	4851	http://gepia.cancer-pku.cn/detail.php?gene=NOTCH1	0.0364109	SDL
Gefitinib	Urethral cancer; Blad	Leukemia, T-Cell	EGFR	1956	EGFR+NOTCH1	NOTCH1	4851	http://gepia.cancer-pku.cn/detail.php?gene=NOTCH1	0.0381146	SDL
Panitumumab	Colorectal cancer	Leukemia, T-Cell	EGFR	1956	EGFR+NOTCH1	NOTCH1	4851	http://gepia.cancer-pku.cn/detail.php?gene=NOTCH1	0.0396495	SDL
Merimepodib	Head and neck squam	Carcinoma, Pancreatic Ductal	EGFR	1956	EGFR+NOTCH1	NOTCH1	4851	http://gepia.cancer-pku.cn/detail.php?gene=NOTCH1	0.0400193	SDL
Merimepodib	Refractory breast can	Carcinoma, Pancreatic Ductal	EGFR	1956	EGFR+NOTCH1	NOTCH1	4851	http://gepia.cancer-pku.cn/detail.php?gene=NOTCH1	0.0407823	SDL
Afatinib	Non-small-cell lung ca	Precursor T-Cell Lymphoblastic Leukemia-Lymphoma	EGFR	1956	EGFR+NOTCH1	NOTCH1	4851	http://gepia.cancer-pku.cn/detail.php?gene=NOTCH1	0.0414984	SDL
Nimotuzumab	Head and neck cancer	Carcinoma, Pancreatic Ductal	EGFR	1956	EGFR+NOTCH1	NOTCH1	4851	http://gepia.cancer-pku.cn/detail.php?gene=NOTCH1	0.0441368	SDL
Lapatinib	Refractory metastatic	Carcinoma, Pancreatic Ductal	EGFR	1956	EGFR+NOTCH1	NOTCH1	4851	http://gepia.cancer-pku.cn/detail.php?gene=NOTCH1	0.045748	SDL
Pertuzumab	Breast cancer	Carcinoma, Pancreatic Ductal	EGFR	1956	EGFR+NOTCH1	NOTCH1	4851	http://gepia.cancer-pku.cn/detail.php?gene=NOTCH1	0.0590941	SDL
Panitumumab	Colorectal cancer	Lymphoma	EGFR	1956	EGFR+NOTCH1	NOTCH1	4851	http://gepia.cancer-pku.cn/detail.php?gene=NOTCH1	0.0627319	SDL
Lidocaine	Anesthesia	Lymphoma	EGFR	1956	EGFR+NOTCH1	NOTCH1	4851	http://gepia.cancer-pku.cn/detail.php?gene=NOTCH1	0.0635375	SDL
Gefitinib	Head and neck cancer	Carcinoma, Pancreatic Ductal	EGFR	1956	EGFR+NOTCH1	NOTCH1	4851	http://gepia.cancer-pku.cn/detail.php?gene=NOTCH1	0.0674305	SDL
Necitumumab	Squamous non-small c	Carcinoma, Pancreatic Ductal	EGFR	1956	EGFR+NOTCH1	NOTCH1	4851	http://gepia.cancer-pku.cn/detail.php?gene=NOTCH1	0.0761895	SDL
Necitumumab	Non-small-cell lung ca	Lymphoma	EGFR	1956	EGFR+NOTCH1	NOTCH1	4851	http://gepia.cancer-pku.cn/detail.php?gene=NOTCH1	0.077943	SDL
Merimepodib	Refractory breast can	Precursor T-Cell Lymphoblastic Leukemia-Lymphoma	EGFR	1956	EGFR+NOTCH1	NOTCH1	4851	http://gepia.cancer-pku.cn/detail.php?gene=NOTCH1	0.0789834	SDL
Lapatinib	Refractory metastatic	Leukemia, T-Cell	EGFR	1956	EGFR+NOTCH1	NOTCH1	4851	http://gepia.cancer-pku.cn/detail.php?gene=NOTCH1	0.0817436	SDL
Afatinib	Non-small-cell lung ca	Carcinoma, Pancreatic Ductal	EGFR	1956	EGFR+NOTCH1	NOTCH1	4851	http://gepia.cancer-pku.cn/detail.php?gene=NOTCH1	0.0832257	SDL
Panitumumab	Colorectal cancer	Carcinoma, Pancreatic Ductal	EGFR	1956	EGFR+NOTCH1	NOTCH1	4851	http://gepia.cancer-pku.cn/detail.php?gene=NOTCH1	0.0896368	SDL
Cetuximab	Colorectal cancer	Lymphoma	EGFR	1956	EGFR+NOTCH1	NOTCH1	4851	http://gepia.cancer-pku.cn/detail.php?gene=NOTCH1	0.0996343	SDL
Merimepodib	Head and neck squam	Precursor T-Cell Lymphoblastic Leukemia-Lymphoma	EGFR	1956	EGFR+NOTCH1	NOTCH1	4851	http://gepia.cancer-pku.cn/detail.php?gene=NOTCH1	0.1365309	SDL
Lapatinib	Refractory metastatic	Precursor T-Cell Lymphoblastic Leukemia-Lymphoma	EGFR	1956	EGFR+NOTCH1	NOTCH1	4851	http://gepia.cancer-pku.cn/detail.php?gene=NOTCH1	0.1459077	SDL
Gefitinib	Head and neck cancer	Precursor T-Cell Lymphoblastic Leukemia-Lymphoma	EGFR	1956	EGFR+NOTCH1	NOTCH1	4851	http://gepia.cancer-pku.cn/detail.php?gene=NOTCH1	0.155283	SDL
Erlotinib	Colon cancer; Glioma	Lymphoma	EGFR	1956	EGFR+NOTCH1	NOTCH1	4851	http://gepia.cancer-pku.cn/detail.php?gene=NOTCH1	0.1576118	SDL
Erlotinib	Pancreatic cancer	Precursor T-Cell Lymphoblastic Leukemia-Lymphoma	EGFR	1956	EGFR+NOTCH1	NOTCH1	4851	http://gepia.cancer-pku.cn/detail.php?gene=NOTCH1	0.159603	SDL
Gefitinib	Head and neck cancer	Lymphoma	EGFR	1956	EGFR+NOTCH1	NOTCH1	4851	http://gepia.cancer-pku.cn/detail.php?gene=NOTCH1	0.1637162	SDL
Nimotuzumab	Head and neck cancer	Precursor T-Cell Lymphoblastic Leukemia-Lymphoma	EGFR	1956	EGFR+NOTCH1	NOTCH1	4851	http://gepia.cancer-pku.cn/detail.php?gene=NOTCH1	0.1669782	SDL
Gefitinib	Cancers	Lymphoma	EGFR	1956	EGFR+NOTCH1	NOTCH1	4851	http://gepia.cancer-pku.cn/detail.php?gene=NOTCH1	0.1767057	SDL
Panitumumab	Locally advanced hea	Precursor T-Cell Lymphoblastic Leukemia-Lymphoma	EGFR	1956	EGFR+NOTCH1	NOTCH1	4851	http://gepia.cancer-pku.cn/detail.php?gene=NOTCH1	0.1964762	SDL
Panitumumab	Colorectal cancer	Precursor T-Cell Lymphoblastic Leukemia-Lymphoma	EGFR	1956	EGFR+NOTCH1	NOTCH1	4851	http://gepia.cancer-pku.cn/detail.php?gene=NOTCH1	0.1973229	SDL
Merimepodib	Head and neck squam	Lymphoma	EGFR	1956	EGFR+NOTCH1	NOTCH1	4851	http://gepia.cancer-pku.cn/detail.php?gene=NOTCH1	0.2035711	SDL
Lapatinib	Breast cancer	Precursor T-Cell Lymphoblastic Leukemia-Lymphoma	EGFR	1956	EGFR+NOTCH1	NOTCH1	4851	http://gepia.cancer-pku.cn/detail.php?gene=NOTCH1	0.2052322	SDL
Merimepodib	Gastric cancer	Leukemia, T-Cell	EGFR	1956	EGFR+NOTCH1	NOTCH1	4851	http://gepia.cancer-pku.cn/detail.php?gene=NOTCH1	0.2107555	SDL
Lapatinib	Refractory metastatic	Lymphoma	EGFR	1956	EGFR+NOTCH1	NOTCH1	4851	http://gepia.cancer-pku.cn/detail.php?gene=NOTCH1	0.2289016	SDL
Cetuximab	Colorectal cancer	Carcinoma, Pancreatic Ductal	EGFR	1956	EGFR+NOTCH1	NOTCH1	4851	http://gepia.cancer-pku.cn/detail.php?gene=NOTCH1	0.2292831	SDL
Gefitinib	Head and neck cancer	Leukemia, T-Cell	EGFR	1956	EGFR+NOTCH1	NOTCH1	4851	http://gepia.cancer-pku.cn/detail.php?gene=NOTCH1	0.2589028	SDL
Gefitinib	Cancers	Precursor T-Cell Lymphoblastic Leukemia-Lymphoma	EGFR	1956	EGFR+NOTCH1	NOTCH1	4851	http://gepia.cancer-pku.cn/detail.php?gene=NOTCH1	0.2627547	SDL
Erlotinib	Pancreatic cancer	Leukemia, T-Cell	EGFR	1956	EGFR+NOTCH1	NOTCH1	4851	http://gepia.cancer-pku.cn/detail.php?gene=NOTCH1	0.2736356	SDL
Lapatinib	Breast cancer	Lymphoma	EGFR	1956	EGFR+NOTCH1	NOTCH1	4851	http://gepia.cancer-pku.cn/detail.php?gene=NOTCH1	0.2814613	SDL
Erlotinib	Non-small-cell lung ca	Lymphoma	EGFR	1956	EGFR+NOTCH1	NOTCH1	4851	http://gepia.cancer-pku.cn/detail.php?gene=NOTCH1	0.297145	SDL
Erlotinib	Pancreatic cancer	Lymphoma	EGFR	1956	EGFR+NOTCH1	NOTCH1	4851	http://gepia.cancer-pku.cn/detail.php?gene=NOTCH1	0.2973886	SDL
Gefitinib	Urethral cancer; Blad	Precursor T-Cell Lymphoblastic Leukemia-Lymphoma	EGFR	1956	EGFR+NOTCH1	NOTCH1	4851	http://gepia.cancer-pku.cn/detail.php?gene=NOTCH1	0.300005	SDL
Necitumumab	Colorectal cancer	Precursor T-Cell Lymphoblastic Leukemia-Lymphoma	EGFR	1956	EGFR+NOTCH1	NOTCH1	4851	http://gepia.cancer-pku.cn/detail.php?gene=NOTCH1	0.3038599	SDL
Pertuzumab	Breast cancer	Lymphoma	EGFR	1956	EGFR+NOTCH1	NOTCH1	4851	http://gepia.cancer-pku.cn/detail.php?gene=NOTCH1	0.305339	SDL
Necitumumab	Squamous non-small c	Lymphoma	EGFR	1956	EGFR+NOTCH1	NOTCH1	4851	http://gepia.cancer-pku.cn/detail.php?gene=NOTCH1	0.3141494	SDL
Merimepodib	Gastric cancer	Precursor T-Cell Lymphoblastic Leukemia-Lymphoma	EGFR	1956	EGFR+NOTCH1	NOTCH1	4851	http://gepia.cancer-pku.cn/detail.php?gene=NOTCH1	0.3183873	SDL
Lapatinib	Breast cancer	Leukemia, T-Cell	EGFR	1956	EGFR+NOTCH1	NOTCH1	4851	http://gepia.cancer-pku.cn/detail.php?gene=NOTCH1	0.322766	SDL
Merimepodib	Refractory breast can	Lymphoma	EGFR	1956	EGFR+NOTCH1	NOTCH1	4851	http://gepia.cancer-pku.cn/detail.php?gene=NOTCH1	0.3337232	SDL
Panitumumab	Locally advanced hea	Carcinoma, Pancreatic Ductal	EGFR	1956	EGFR+NOTCH1	NOTCH1	4851	http://gepia.cancer-pku.cn/detail.php?gene=NOTCH1	0.3416839	SDL
Merimepodib	Gastric cancer	Carcinoma, Pancreatic Ductal	EGFR	1956	EGFR+NOTCH1	NOTCH1	4851	http://gepia.cancer-pku.cn/detail.php?gene=NOTCH1	0.3597037	SDL
Panitumumab	Locally advanced hea	Leukemia, T-Cell	EGFR	1956	EGFR+NOTCH1	NOTCH1	4851	http://gepia.cancer-pku.cn/detail.php?gene=NOTCH1	0.3665653	SDL
Panitumumab	Locally advanced hea	Lymphoma	EGFR	1956	EGFR+NOTCH1	NOTCH1	4851	http://gepia.cancer-pku.cn/detail.php?gene=NOTCH1	0.3785937	SDL
Erlotinib	Non-small-cell lung ca	Carcinoma, Pancreatic Ductal	EGFR	1956	EGFR+NOTCH1	NOTCH1	4851	http://gepia.cancer-pku.cn/detail.php?gene=NOTCH1	0.3895054	SDL
Erlotinib	Non-small-cell lung ca	Leukemia, T-Cell	EGFR	1956	EGFR+NOTCH1	NOTCH1	4851	http://gepia.cancer-pku.cn/detail.php?gene=NOTCH1	0.3911063	SDL
Nimotuzumab	Head and neck cancer	Leukemia, T-Cell	EGFR	1956	EGFR+NOTCH1	NOTCH1	4851	http://gepia.cancer-pku.cn/detail.php?gene=NOTCH1	0.3976862	SDL
Gefitinib	Urethral cancer; Blad	Carcinoma, Pancreatic Ductal	EGFR	1956	EGFR+NOTCH1	NOTCH1	4851	http://gepia.cancer-pku.cn/detail.php?gene=NOTCH1	0.4072126	SDL
Lapatinib	Breast cancer	Carcinoma, Pancreatic Ductal	EGFR	1956	EGFR+NOTCH1	NOTCH1	4851	http://gepia.cancer-pku.cn/detail.php?gene=NOTCH1	0.4276918	SDL
Erlotinib	Pancreatic cancer	Carcinoma, Pancreatic Ductal	EGFR	1956	EGFR+NOTCH1	NOTCH1	4851	http://gepia.cancer-pku.cn/detail.php?gene=NOTCH1	0.4477807	SDL
Vandetanib	Solid tumours	Leukemia, T-Cell	EGFR	1956	EGFR+NOTCH1	NOTCH1	4851	http://gepia.cancer-pku.cn/detail.php?gene=NOTCH1	0.457649	SDL
Pertuzumab	Breast cancer	Leukemia, T-Cell	EGFR	1956	EGFR+NOTCH1	NOTCH1	4851	http://gepia.cancer-pku.cn/detail.php?gene=NOTCH1	0.4761516	SDL
Merimepodib	Gastric cancer	Lymphoma	EGFR	1956	EGFR+NOTCH1	NOTCH1	4851	http://gepia.cancer-pku.cn/detail.php?gene=NOTCH1	0.4798495	SDL
Merimepodib	Refractory breast can	Leukemia, T-Cell	EGFR	1956	EGFR+NOTCH1	NOTCH1	4851	http://gepia.cancer-pku.cn/detail.php?gene=NOTCH1	0.5068601	SDL
Lidocaine	Anesthesia	Precursor T-Cell Lymphoblastic Leukemia-Lymphoma	EGFR	1956	EGFR+NOTCH1	NOTCH1	4851	http://gepia.cancer-pku.cn/detail.php?gene=NOTCH1	0.5127445	SDL
Necitumumab	Squamous non-small c	Leukemia, T-Cell	EGFR	1956	EGFR+NOTCH1	NOTCH1	4851	http://gepia.cancer-pku.cn/detail.php?gene=NOTCH1	0.5362979	SDL
Necitumumab	Squamous non-small c	Precursor T-Cell Lymphoblastic Leukemia-Lymphoma	EGFR	1956	EGFR+NOTCH1	NOTCH1	4851	http://gepia.cancer-pku.cn/detail.php?gene=NOTCH1	0.5707953	SDL
Nimotuzumab	Head and neck cancer	Lymphoma	EGFR	1956	EGFR+NOTCH1	NOTCH1	4851	http://gepia.cancer-pku.cn/detail.php?gene=NOTCH1	0.5800356	SDL
Gefitinib	Cancers	Leukemia, T-Cell	EGFR	1956	EGFR+NOTCH1	NOTCH1	4851	http://gepia.cancer-pku.cn/detail.php?gene=NOTCH1	0.581861	SDL
Gefitinib	Urethral cancer; Blad	Lymphoma	EGFR	1956	EGFR+NOTCH1	NOTCH1	4851	http://gepia.cancer-pku.cn/detail.php?gene=NOTCH1	0.5944808	SDL
Vandetanib	Solid tumours	Precursor T-Cell Lymphoblastic Leukemia-Lymphoma	EGFR	1956	EGFR+NOTCH1	NOTCH1	4851	http://gepia.cancer-pku.cn/detail.php?gene=NOTCH1	0.6020115	SDL
Erlotinib	Non-small-cell lung ca	Precursor T-Cell Lymphoblastic Leukemia-Lymphoma	EGFR	1956	EGFR+NOTCH1	NOTCH1	4851	http://gepia.cancer-pku.cn/detail.php?gene=NOTCH1	0.6160136	SDL
Vandetanib	Solid tumours	Carcinoma, Pancreatic Ductal	EGFR	1956	EGFR+NOTCH1	NOTCH1	4851	http://gepia.cancer-pku.cn/detail.php?gene=NOTCH1	0.6868177	SDL
Merimepodib	Head and neck squam	Leukemia, T-Cell	EGFR	1956	EGFR+NOTCH1	NOTCH1	4851	http://gepia.cancer-pku.cn/detail.php?gene=NOTCH1	0.6949075	SDL
Necitumumab	Colorectal cancer	Carcinoma, Pancreatic Ductal	EGFR	1956	EGFR+NOTCH1	NOTCH1	4851	http://gepia.cancer-pku.cn/detail.php?gene=NOTCH1	0.7002856	SDL
Erlotinib	Colon cancer; Glioma	Precursor T-Cell Lymphoblastic Leukemia-Lymphoma	EGFR	1956	EGFR+NOTCH1	NOTCH1	4851	http://gepia.cancer-pku.cn/detail.php?gene=NOTCH1	0.7375828	SDL
Necitumumab	Colorectal cancer	Leukemia, T-Cell	EGFR	1956	EGFR+NOTCH1	NOTCH1	4851	http://gepia.cancer-pku.cn/detail.php?gene=NOTCH1	0.7632419	SDL
Pertuzumab	Breast cancer	Precursor T-Cell Lymphoblastic Leukemia-Lymphoma	EGFR	1956	EGFR+NOTCH1	NOTCH1	4851	http://gepia.cancer-pku.cn/detail.php?gene=NOTCH1	0.8403559	SDL

Supplementary Table 4: Mapping between tumors and cancer types

Repositioning of the Cancer	Cancer Type	Cancer Type Name
Familial medullary thyroid carcinoma	THCA	Thyroid carcinoma
Hereditary Breast and Ovarian Cancer Syndrome	BRCA;OV	Breast invasive carcinoma;Ovarian serous cystadenocarcinoma
Ovarian Carcinoma	OV	Ovarian serous cystadenocarcinoma
Melanoma-Pancreatic Cancer Syndrome	SKCM;PAAD	Skin Cutaneous Melanoma;Pancreatic adenocarcinoma
Small cell carcinoma of lung	LUAD	Lung adenocarcinoma
Carcinoma,Pancreatic Ductal	PAAD	Pancreatic adenocarcinoma
Leukemia, T-Cell	LAML	Acute Myeloid Leukemia
Lymphoma	NULL	NULL
Precursor T-Cell Lymphoblastic Leukemia-Lymphoma	LAML	Acute Myeloid Leukemia
Retinoblastma	NULL	NULL

Supplementary Table 5. Primer sequences used in this study

Gene	Sequence
CDKN2A-Forward	TCTGGAGTGAGCACTCACGC
CDKN2A-Reverse	GCTTGTCATGAAGTCGACAGCTTT
GAPDH1-Forward	ATGACATCAAGAAGGTGGTG
GAPDH1-Reverse	CATACCAGGAAATGAGCTTG
